# Bacterial Fatty Acids Enhance Recovery from the Dauer Larva in *Caenorhabditis elegans*

**DOI:** 10.1371/journal.pone.0086979

**Published:** 2014-01-24

**Authors:** Tiffany K. Kaul, Pedro Reis Rodrigues, Ifedayo V. Ogungbe, Pankaj Kapahi, Matthew S. Gill

**Affiliations:** 1 Department of Metabolism & Aging, The Scripps Research Institute – Scripps Florida, Jupiter, Florida, United States of America; 2 Buck Institute for Research on Aging, Novato, California, United States of America; University of North Carolina, United States of America

## Abstract

The dauer larva is a specialized dispersal stage in the nematode *Caenorhabditis elegans* that allows the animal to survive starvation for an extended period of time. The dauer does not feed, but uses chemosensation to identify new food sources and to determine whether to resume reproductive growth. Bacteria produce food signals that promote recovery of the dauer larva, but the chemical identities of these signals remain poorly defined. We find that bacterial fatty acids in the environment augment recovery from the dauer stage under permissive conditions. The effect of increased fatty acids on different dauer constitutive mutants indicates a role for insulin peptide secretion in coordinating recovery from the dauer stage in response to fatty acids. These data suggest that worms can sense the presence of fatty acids in the environment and that elevated levels can promote recovery from dauer arrest. This may be important in the natural environment where the dauer larva needs to determine whether the environment is appropriate to support reproductive growth following dauer exit.

## Introduction

During development, the nematode *C. elegans* acutely senses its environment by integrating signals pertaining to food availability, temperature, and population density in order to determine whether conditions are appropriate to support normal larval development and subsequent progeny production [1]. If conditions are deemed unsuitable, developing first-stage larvae are able to change their developmental program to enter an alternate third larval stage called the dauer larva, which is non-feeding, non-reproducing, and long-lived [2], [3]. Once the dauer larva encounters environmental conditions that are more conducive to growth, the animal is able to exit this diapause stage and develop into a fertile adult.

The dauer pheromone, temperature, and food availability are the major signals that regulate dauer entry and exit [1]. In early studies of dauer formation, Riddle and colleagues determined that entry into dauer is influenced by a pheromone which is produced by the worm and whose concentration reflects population density [4]–[6]. In contrast, recovery from dauer could be stimulated by a food signal derived from bacteria [7]. Recent studies have determined that the dauer pheromone is composed of a complex mixture of small molecules called ascarosides [8]. However, little is known about the chemical nature of the bacterial food signal [7].

Genetic analysis of dauer formation has delineated a complex set of interacting pathways that act to integrate environmental signals and coordinate the organismal response to proceed either with reproductive growth or arrest in the dauer state [9]. Multiple chemosensory inputs feed into TGF-β-like and insulin-like signaling pathways, which ultimately converge on the nuclear receptor DAF-12. Laser ablation studies, along with analysis of mutants with defective chemosensation, have defined a subset of sensory neurons that are involved in dauer formation [10]. Of these, ASI and ASJ play a major role in dauer entry, while ASJ is also critical for dauer recovery [11], [12]. These neurons also express the guanylate cyclase DAF-11 [13], which has been shown to influence expression and availability of the TGF-β-like peptide DAF-7 [14] and the insulin peptide DAF-28 [15]. Another insulin peptide, INS-6, is also expressed in ASI and ASJ [12], [16] and is required for both dauer formation and dauer recovery [12].

In the laboratory, *C. elegans* is maintained under monoxenic conditions with *E. coli* OP50 being the typical bacterial strain used as a food source. The bacterial diet provides the major source of fatty acids, although *C. elegans* is able to synthesize fatty acids *de novo* [17]–[19]. During the exponential growth phase the majority of bacterial fatty acids are saturated or monounsaturated. However, when bacteria enter stationary phase they induce the enzyme cyclopropane fatty acyl phospholipid synthase (*cfa)* as part of the stringent response [20]. *cfa* catalyzes the formation of cyclopropane fatty acids (CFA) from monounsaturated fatty acids (MUFA), which are thought to help the bacteria cope with increasing acidity [21]. Typically, nematode growth medium plates are seeded with a small volume of an overnight bacterial culture and then left for a few days for the bacterial lawn to develop. Thus, worms under routine culture conditions ingest a significant amount of cyclopropane fatty acids, which can be detected in their triglyceride and phospholipid fractions [17]. Despite this class of fatty acids comprising up to 35% of their total fatty acids [18], little is known about their metabolic fate or biological function in worms.

In order to understand the role of CFA in worms, we took advantage of an *E. coli* K12 strain that lacks the *cfa* enzyme [22] and therefore does not synthesize CFA even during stationary phase. We found that recovery from dauer was enhanced in the presence of *cfa* mutant bacteria, and this increase in recovery did not appear to be due to the absence of CFA *per se,* but was related to the corresponding increase in MUFA in these bacteria. The increase in dauer recovery appeared to be mediated by the ASJ sensory neuron, because the effect required the guanylate cyclase DAF-11, the insulin peptide INS-6, and was absent in animals in which ASJ was genetically ablated. Moreover, dauer recovery could be augmented by exogenous saturated and monounsaturated fatty acids in the absence of bacteria, suggesting that worms are responding to fatty acids in the environment and not a secondary metabolite produced by the bacteria. We propose that the ability of dauers to sense free fatty acids could reflect a mechanism by which the animal senses the nutritional content of a food source and adjusts its recovery accordingly.

## Materials and Methods

### Chemicals

Phytomonic acid (C18 CFA), palmitoleic acid (C16∶1), palmitic acid (C16∶0), oleic acid (C18∶1n9), and stearic acid (C18∶0) were obtained from Cayman Chemical (MI). Lauric acid (C12∶0), myristic acid (C14∶0), vaccenic acid (C18∶1n7), elaidic acid (*trans-*C18∶1n9), linoleic acid (C18∶2n6), arachidonic acid (C20∶4n6), and eicosapentaenoic acid (C20∶5n3) were from Nu-Chek Prep, Inc. (MN). All solvents were of GC-MS grade and all other reagents were of the highest grade available.

### Bacterial Strains and Maintenance

The parent *E. coli* K12 strain BW25113 and the *cfa* deletion mutant JW1653 were part of the Keio mutant collection [22]. Bacteria were grown in Luria broth with 35 µg/mL kanamycin supplementation for JW1653.

### *C. elegans* Strains and Maintenance

*C. elegans* strains were maintained as previously described [23]. The following strains were obtained from the *Caenorhabditis* Genetics Center at the University of Minnesota: Bristol N2 (wild-type), *daf-2(e1368), daf-2(e1371), daf-2(e1370), daf-2(m41), daf-7(e1372), daf-11(m47),* and *unc-31(e928). ins-6(tm2416)* and *daf-28(tm2308)* were obtained from Dr. Shohei Mitani at the National Bioresource Project at Tokyo Women’s Medical University School of Medicine. The *ins-6* and *daf-28* mutations were each crossed into *daf-2(e1371)* and the resulting double mutants were genotyped to confirm the presence of the mutant alleles. The double mutants were then backcrossed into *daf-2(e1371)* a further two times. *daf-2(e1368); jxEx18[ofm-1::gfp], daf-2(e1368); jxEx102[pQZ37(trx-1::ICE) ofm-1::gfp]* and *daf-2(e1368); jxEx100[pQZ37(trx-1::ICE) ofm-1::gfp]* were a kind gift from Dr. Joy Alcedo. Worms were conditioned on each bacterial strain for three generations prior to analysis.

### FAME Analysis

Analysis of fatty acid methyl esters (FAMEs) in bacteria and worms was performed as previously described with minor modifications [17]. For bacteria, 3 mL of an overnight culture was centrifuged and the bacterial pellet collected. For worms, 50 gravid adults were picked into S-basal, washed in 3 volumes of S-basal, and snap frozen in a 10 µL volume. To generate FAMEs, samples were re-suspended in 1 mL methanol containing 2.5% H_2_SO_4_ in glass tubes and incubated in a water bath at 80°C for 1 h. After addition of 1.5 mL water and 200 µL hexane, the samples were vortexed twice for 1 min and the hexane layer transferred to a glass vial until analysis.

1 µL FAMEs in hexane were analyzed using an Agilent 7890A GC 240 MS ion trap system operating in split mode (split ratio 10∶1) with a VF-5ms capillary column (30 m×0.25 mm i.d., 5% phenyl-95% methyl polysiloxine, 0.25 µm film thickness; Varian, Inc., Walnut Creek, CA). The GC conditions were: injector 240°C, initial column temperature 120°C for 0.1 min, temperature ramp 15°C per min to 190°C, 2°C ramp to 220°C, 20°C per min to 250°C, 7 min hold, with a flow rate of 1.1 mL/min. FAMEs were analyzed in EI mode and data were collected using Agilent MS workstation software system control version 7.0.0.

### Dauer Recovery Assays

Compounds were re-suspended in ethanol to a final concentration of 20 mM. For dose range experiments serial dilutions were made to yield 10, 5, 2, 1, and 0.5 mM. 20 µL of each working solution of compound was added to 130 µL water before being spotted onto a 4 mL NGM plate. Equal distribution of the compound throughout the agar was assumed to yield final concentrations of 50, 25, 10, 5, and 2.5 µM.

For the dauer recovery assays, eggs from a synchronous lay were transferred to the appropriate plates and incubated at the restrictive temperature. After 3 days, dauers were selected by treatment with 1% sodium dodecyl sulfate (SDS) for 15 minutes, transferred to a fresh plate, and incubated at the permissive temperature for 24 h. Recovery was scored by counting dauer and non-dauer animals after treatment with 1% SDS for 15 min. Recovery assays in the absence of bacteria were carried out on NGM plates supplemented with ampicillin to prevent growth of trace amounts of bacteria that may have been carried over following SDS treatment.

To generate wild type (N2) starvation-induced dauers, worms were grown in liquid culture. Starved L1s from a 5 cm NGM plate were inoculated into 100 mL of S-media in a 1 L flask containing 10 mL of concentrated K12 bacteria and incubated at 25°C in a shaker incubator at 150 rpm. After 3–4 days, when the original food source was depleted, an additional 10 mL of concentrated bacteria was added, and worms were grown for a further 3–4 days. Worms were harvested by centrifugation and dauers were isolated following selection in 1% SDS.

### Statistical Analysis

The number of dauers and non-dauers were scored from triplicate plates for each trial and trials set up on different days were deemed to be biological replicates. The percentage of animals that recovered from dauer was calculated for each trial. Data are presented as median, interquartile range and min/max and were analyzed by one way ANOVA with Sidak’s multiple comparisons test for k ≥3 groups or by Student’s t-test for k = 2 groups. Statistical analysis was performed using GraphPad Prism. Raw data, including the number of worms scored per trial, as well as the conditions for each dauer recovery assay, can be found in Table S1.

## Results

### Worms Accumulate Cyclopropane Fatty Acids from their Diet

**Figure 1:**
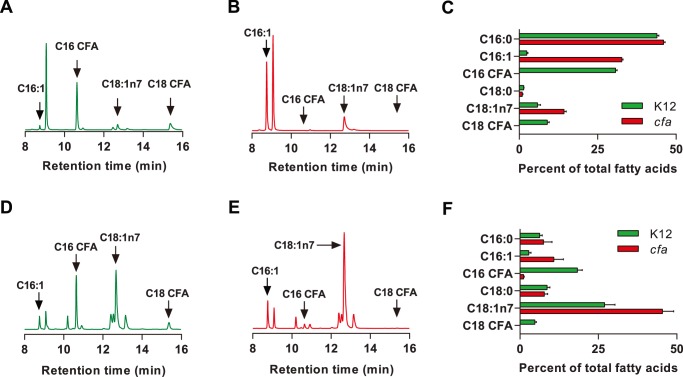
Worms grown on *cfa* bacteria have reduced cyclopropane fatty acids and elevated monounsaturated fatty acids. Representative gas chromatograms showing fatty acids from (**A**) *E. coli* K12 bacteria and (**B**) *cfa* bacteria. (**C**) The amount of each bacterial fatty acid expressed as a percent of the total area under the curve for K12 and *cfa* bacteria. Data are presented as mean+sd for 5 independent bacterial samples. Representative gas chromatograms of fatty acids from *daf-2(e1368)* adult worms grown on (**D**) *E. coli* K12 and (**E**) *cfa* bacteria. (**F**) The amount of each worm fatty acid expressed as a percent of the total area under the curve for *daf-2(e1368)* adult worms grown on K12 and *cfa* bacteria. Data are presented as mean+sd for 5 independent worm samples.

*E. coli* K12 bacteria produced both C16 and C18 CFA (Figure 1A), while in *cfa* mutant bacteria the absence of CFA was associated with a concomitant rise in C16∶1 and C18∶1 MUFA (Figure 1B & C). Worms grown on *E. coli* K12 accumulated C16 and C18 CFA (Figure 1D) as has been observed on *E. coli* OP50 [18]. In contrast, worms grown on *cfa* bacteria lacked C18 CFA and had greatly reduced levels of C16 CFA (Figure 1E), along with an increase in the amount of C16∶1 and C18∶1 MUFA (Figure 1F).

### Bacterial Fatty Acids Influence Dauer Recovery

**Figure 2:**
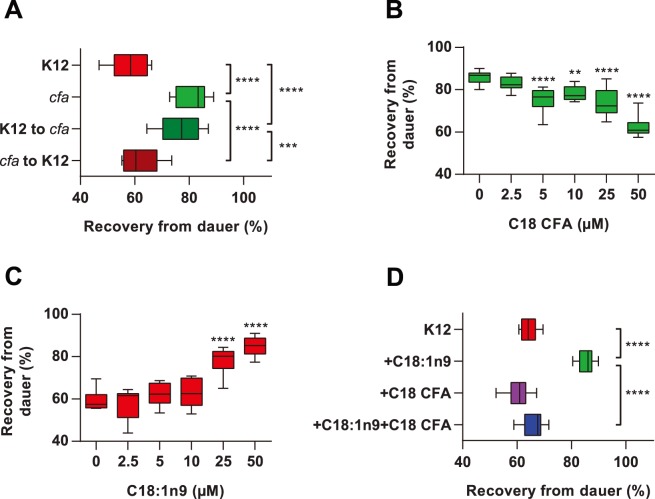
Bacterial fatty acids influence recovery in *daf-2(e1368)* dauers at 20°C. (**A**) *daf-2* dauers showed increased recovery on *cfa* bacteria at 20°C irrespective of whether they were raised on *E. coli* K12 or *cfa* bacteria. (**B**) *daf-2* dauers showed decreased recovery on *cfa* bacteria in the presence of exogenous C18 CFA at 20°C. (**C**) *daf-2* dauers showed increased recovery on K12 bacteria when supplemented with exogenous C18∶1n9. (**D**) The increase in dauer recovery in the presence of 50 µM C18∶1n9 was prevented when worms were incubated with 50 µM C18∶1n9+50 µM C18 CFA. (****p<0.0001, ***p<0.001, **p<0.01 for indicated pairwise comparisons).

We found that *cfa* bacteria did not affect the fraction of *daf-2(e1368)* animals that formed dauers when grown at 25°C (data not shown). However, *daf-2(e1368)* dauers formed at 25°C on *cfa* bacteria showed increased recovery after 24 h at 20°C compared with dauers formed on K12 (Figure 2A). We considered that the presence of CFA in the worm could be slowing down dauer recovery, so we grew dauers on K12 and *cfa* bacteria and shifted them to the opposite condition during recovery. Notably, dauers that had developed on K12 bacteria, but recovered in the presence of *cfa* bacteria, showed the same increase in recovery as dauers raised and recovered on *cfa* (Figure 2A). Conversely, dauers formed on *cfa* bacteria and recovered on K12 bacteria showed the same recovery as animals raised and recovered on K12 alone (Figure 2A). These data suggest that dauers sense their bacterial environment and alter their recovery accordingly.

We hypothesized that the difference in fatty acid composition between K12 and *cfa* bacteria was influencing the recovery rates, i.e. reduced CFA or increased MUFA in *cfa* bacteria. Supplementation of *cfa* bacteria with phytomonic acid, a C18 CFA from *Lactobacillus arabinosus,* reduced dauer recovery in *daf-2(e1368)* mutants in a dose dependent manner (Figure 2B), suggesting that increased CFA inhibits dauer recovery. Conversely, supplementation of *E. coli* K12 bacteria with C18∶1n9 dose-dependently promoted dauer recovery (Figure 2C) as did treatment with C16∶1 or C18∶1n7 (Figure S1A). Interestingly, supplementation with saturated fatty acids (C16∶0 or C18∶0) also dose-dependently augmented dauer recovery (Figure S1B). Neither *cfa* bacteria nor exogenous C18∶1n9 promoted dauer exit in dauers maintained at the restrictive temperature in the presence of food (Figure S1C). To determine the relative importance of high MUFA or low CFA we examined the effect of co-exposure to the fatty acids in the presence of K12 bacteria. Supplementation of K12 bacteria with CFA did not reduce recovery below K12 alone, but the addition of CFA+MUFA did prevent the increased recovery associated with MUFA treatment alone (Figure 2D). This suggests that recovery is influenced by the relative amounts of MUFA and CFA in the environment during the recovery period.

### Dauer Recovery in Response to Fatty Acids Requires the Guanylate Cyclase *daf-11* and the Insulin Peptide *ins-6*

**Figure 3:**
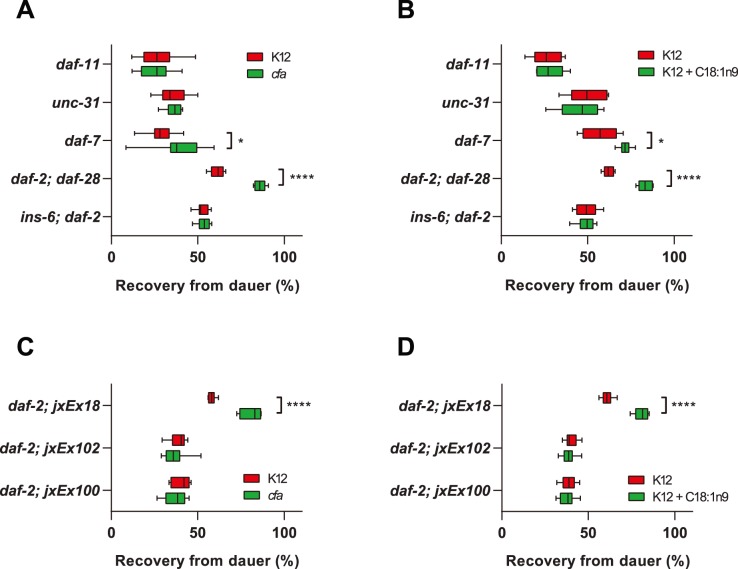
Increased dauer recovery in response to *cfa* bacteria and increased MUFA requires the guanylate cyclase *daf-11* and the insulin peptide *ins-6.* (**A**) Dauer recovery was enhanced on *cfa* bacteria compared with K12 bacteria in *daf-7* and *daf-2; daf-28* mutants, but not *daf-11, unc-31* or *ins-6; daf-2* mutants. (**B**) Dauer recovery was enhanced on K12 bacteria +50 µM C18∶1n9 compared with K12 bacteria in *daf-7* and *daf-2; daf-28* mutants, but not *daf-11, unc-31* or *ins-6; daf-2* mutants. (**C**) *cfa* bacteria had no effect on dauer recovery in *daf-2(e1368)* dauers in which the ASJ sensory neuron was genetically ablated (*daf-2; jxEx102* and *daf-2; jxEx100*) compared with the control line (*daf-2; jxEx18).* (**D**) Supplementation of K12 bacteria with 50 µM C18∶1n9 had no effect on dauer recovery in *daf-2(e1368)* dauers in which the ASJ sensory neuron was genetically ablated (*daf-2; jxEx102* and *daf-2; jxEx100*) compared with the control line (*daf-2; jxEx18).* (****p<0.0001, **p<0.01, *p<0.05 for indicated pairwise comparisons).

In order to determine the mechanism by which *cfa* bacteria and exogenous fatty acids promote recovery, we examined recovery from dauer in a number of other mutants that affect dauer formation. First, we determined that the increase in recovery was not specific to the *daf-2(e1368)* mutants because other *daf-2* alleles showed increased recovery on *cfa* bacteria and on K12 bacteria supplemented with C18∶1n9 (Figure S2). Since exposure of dauers to elevated levels of C18∶1n9 was sufficient to influence dauer exit, we hypothesized that fatty acids could be sensed at the level of the sensory neurons, of which, the ASJ neuron has been shown to be important for dauer recovery [11], [12]. The guanylate cyclase, *daf-11*, is expressed in the ASJ neuron and regulates dauer formation downstream of chemosensory receptors [13]. *daf-11(m47)* dauers did not show enhanced recovery in the presence of *cfa* bacteria or 50 µM C18∶1n9, indicating that the signal generated by bacterial fatty acids requires a functional guanylate cyclase and cGMP signaling (Figure 3A & B).

*daf-11* has been placed genetically upstream of neuropeptide release [14], [24] and thus *cfa* bacteria and high C18∶1n9 could be promoting dauer recovery by influencing TGF-β or insulin peptide secretion. In support of this hypothesis, we found that recovery from dauer was not enhanced in *unc-31(e928)* mutants (Figure 3A & B), which have a general defect in neurosecretion. Dauer recovery was enhanced in *daf-7(e1372)* mutants suggesting that the TGF-β pathway is not required for this phenotype. To test if insulin-like peptides were involved in dauer exit we examined the effect of *daf-28* and *ins-6* deletions on the *daf-2* recovery phenotype. Loss of *daf-28,* an insulin peptide important for dauer entry, in the *daf-2(e1371)* background did not affect dauer recovery in response to *cfa* bacteria or C18∶1n9 (Figure 3A & B). However, *ins-6; daf-2* double mutants failed to show increased recovery on *cfa* bacteria or C18∶1n9 (Figure 3A & B) suggesting that elevated bacterial fatty acids promotes dauer recovery via increased *ins-6* signaling. To confirm that the response to fatty acids was being mediated by ASJ we examined dauer recovery in *daf-2* mutants that lack ASJ due to the expression of caspase [12]. In two independent transgenic lines we observed a failure to respond to either *cfa* bacteria (Figure 3C) or exogenous C18∶1n9 (Figure 3D), while non-transgenic siblings were fully responsive (Figure S3).

### Fatty Acids Promote Recovery in the Absence of Bacteria

**Figure 4:**
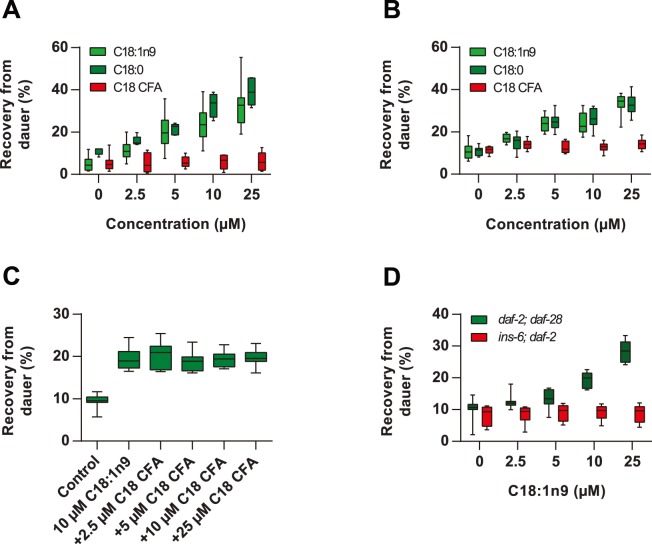
Exogenous fatty acids promote dauer recovery in the absence of bacteria. (**A**) Exogenous C18∶1n9 and C18∶0, but not C18 CFA, promoted recovery of *daf-2(e1368)* dauers in the absence of bacteria. (C18∶1n9 p<0.0001 for 0 vs 5 µM, 0 vs 10 µM and 0 vs 25 µM; C18∶0 p<0.01 for 0 vs 5 µM, p<0.0001 for 0 vs 10 µM and 0 vs 25 µM; C18 CFA all p>0.05)**.** (**B**) Exogenous C18∶1n9 and C18∶0, but not C18 CFA, promoted recovery of starvation-induced wild type dauers in the absence of bacteria. (C18∶1n9 p<0.01 for 0 vs 2.5 µM, p<0.0001 for 0 vs 5 µM, 0 vs 10 µM and 0 vs 25 µM; C18∶0 p<0.0001 for 0 vs 5 µM, 0 vs 10 µM and 0 vs 25 µM; C18 CFA p<0.05 for 0 vs 25 µM)**.** (**C**) C18 CFA did not inhibit the increased recovery of dauers arising from exposure to C18∶1n9 in the absence of bacteria. Dauers were recovered in the absence of fatty acids (Control), or in the presence of 10 µM C18∶1n9 with and without increasing concentrations of C18 CFA. (p<0.0001 for Control vs 10 µM C18∶1n9+ all doses of C18 CFA). (**D**) C18∶1n9 promoted dauer recovery in *daf-2; daf-28*, but not *ins-6; daf-2* dauers, in the absence of bacteria. (*daf-2; daf-28* p<0.0001 for 0 vs 10 µM and 0 vs 25 µM; *ins-6; daf-2*, all p>0.05).

Dauer larvae do not feed and thus the increased recovery observed in the presence of *cfa* bacteria or K12 supplemented with exogenous fatty acids suggests that they are sensing and responding to fatty acids in the environment. We therefore examined dauer recovery in the absence of the bacterial food source, but in the presence of increasing concentrations of fatty acids. Both saturated and monounsaturated fatty acids promoted dauer recovery in *daf-2(e1368)* mutants at 20°C in the absence of bacteria, while C18 CFA had no effect (Figure 4A and S4A). Short chain saturated fatty acids (C12∶0 and C14∶0) and *trans*-C18∶1n9 also promoted dauer recovery, while C18∶2 was less effective and C20 polyunsaturated fatty acids had no effect (Figure S4B). Wild-type starvation-induced dauers also showed increased recovery from dauer in the presence of C18∶0 and C18∶1, but did not respond to C18 CFA (Figure 3B). Interestingly, the presence of C18 CFA during recovery in the absence of bacteria did not suppress the MUFA induced recovery (Figure 4C), which is in contrast to the observation in the presence of bacteria (Figure 2D). Importantly, we found that *daf-2; daf-28* mutants remained responsive to exogenous C18∶1n9 in the absence of bacteria while *ins-6; daf-2* mutants did not show increased recovery on fatty acids alone confirming a role for *ins*-*6* under these conditions (Figure 4D). Together these data suggest that free fatty acids in the environment augment dauer recovery under permissive conditions.

## Discussion

Under standard laboratory conditions, *C. elegans* is fed a bacterial diet that contains cyclopropane fatty acids, which ultimately will comprise around 35% of the nematode’s complement of fatty acids [18]. In this study we set out to determine the role of CFA in the worm by examining the effect of a diet lacking CFA using an *E. coli* deletion strain that lacks the enzyme cyclopropane fatty acyl phospholipid synthase (*cfa*). Ultimately, we did not identify a role for CFA in worms, but we did uncover a role for free fatty acids in promoting recovery from the dauer stage. Using GC-MS, we confirmed the absence of CFA in the bacterial deletion strain and found that worms grown on this bacterial strain also lack CFA. An additional consequence of the lack of CFA was a reciprocal increase in MUFA in both bacteria and worms. Although there was no obvious effect of the lack of CFA on growth and development, we observed that dauer recovery was enhanced in animals raised on *cfa* bacteria.

The dauer recovery phenotype appeared to be a chemosensory response to the bacteria because dauers formed on K12 bacteria contained CFA, but showed the same enhanced recovery on *cfa* bacteria as dauers that were both raised and recovered on *cfa* bacteria. We also demonstrated that this recovery phenotype could be recapitulated by the addition of exogenous fatty acids to the K12 bacteria or C18 CFA to the *cfa* mutant strain. Since the dauer is non-feeding, these data suggested that the difference in fatty acid composition between K12 and *cfa* bacteria can be detected by dauers and that the animal uses this information to adjust its recovery accordingly.

A sensory response to exogenous fatty acids is also supported by the fact that the dauer recovery phenotype was dependent on the guanylate cyclase *daf-11* and the insulin peptide *ins-6*. *daf-11* is expressed in a subset of sensory neurons, including ASJ, and acts to integrate environmental chemosensory inputs into the TGF-β and insulin signaling pathways. *daf-11* has been shown to be involved in regulating the expression of *daf-7* and *daf-28* during entry into dauer [14], [15] as well as insulin peptide expression in adult animals [24]. This suggests a general role in neuropeptide release in response to environmental chemosensory inputs. A neurosecretory consequence of fatty acid exposure was implied by the failure to enhance recovery in *unc-31* mutants which have aberrant dense core vesicle docking [25], [26]. There appears to be a degree of specificity in the neuropeptide response to fatty acids because *daf-7* mutants responded to *cfa* bacteria and C18∶1n9, as did *daf-2;daf-28* mutants that lack an insulin peptide. However, loss of *ins-6* in the *daf-2* background failed to show increased recovery. This is consistent with the important role of INS-6 in dauer recovery previously described by Cornils *et al* [12]. Although ASJ is important for dauer recovery [11], [12], other sensory neurons have been shown to play a role in this process including AWC and IL2 [27]. A major role for ASJ in the response to fatty acids was confirmed using a strain in which the ASJ was genetically ablated [12], and which did not respond to *cfa* bacteria or exogenous fatty acids.

**Figure 5:**
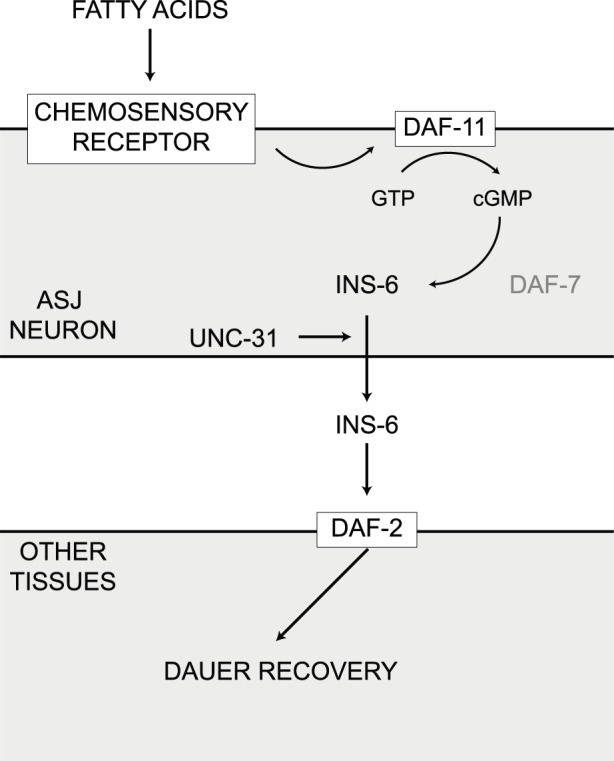
Model for the role of fatty acids in enhancing dauer recovery. Fatty acids interact with an as yet unidentified chemosensory receptor to activate the guanylate cyclase DAF-11. Activation of guanylate cyclase stimulates cGMP production and promotes INS-6 secretion from the ASJ sensory neuron. INS-6 acts on target tissues through the insulin/IGF-I receptor DAF-2 to enhance dauer recovery.

Since *daf-11* functions downstream of chemosensory G-protein coupled receptors (GPCRs) [10] and increased recovery in response to MUFA requires *daf-11*, it is possible that the fatty acid signal is being detected by a GPCR that signals via G-proteins to influence DAF-11 activity (Figure 5). The *C. elegans* genome contains a significant number of GPCRs, but the ligands are known for very few [28]. In mammals, de-orphanization of GPCRs has revealed a number of receptors that respond to free fatty acids of differing carbon chain lengths [29]. Bacteria that lack CFA have a corresponding increase in MUFA with no change in the fraction of saturated fatty acids, suggesting that the putative receptor is responding to the increased MUFA. However, we also found that supplementation with saturated fatty acids worked equally well in promoting dauer recovery. This suggests that bacterial fatty acids could be interacting with a single chemosensory receptor that recognizes both saturated and unsaturated fatty acids or multiple receptors that respond to specific fatty acids.

In this study, we used temperature sensitive mutants to induce dauer formation at the restrictive temperature and initiated dauer recovery by downshifting to a permissive temperature. We did not observe any increase in dauer recovery in dauers that were exposed to *cfa* bacteria or C18∶1n9 at the restrictive temperature, suggesting that the bacterial fatty acids alone were not sufficient to promote recovery under these conditions. However, we did observe a dose dependent increase in dauer recovery in response to both C18 saturated and monounsaturated fatty acids at 20°C in the absence of bacteria, while C18 CFA alone had no effect. Importantly, we also found that wild type, starvation-induced dauers also showed increased recovery in the presence of fatty acids, but the absence of bacteria. Interestingly, other fatty acids that are present in bacteria also promoted recovery in *daf-2* dauers, while long chain polyunsaturated fatty acids, which are not synthesized by bacteria, had no effect. In combination, these data suggest that free fatty acids, derived from the bacteria, could be acting as a weak food signal.

Dauer recovery, like dauer entry, is influenced by temperature, pheromone, and a food signal. In contrast to the progress that has been made in defining the molecular composition of the dauer pheromone [8], the exact chemical nature of the food signal that promotes dauer recovery remains elusive. In an initial characterization, Golden and Riddle determined that the food signal was present in yeast extract and other bacteriological media, was water soluble, could not be extracted into organic solvents, and may be a nucleoside [7]. This would indicate that if fatty acids are being sensed by the worm then they are unlikely to be the same food signal identified by Golden and Riddle and suggests that there may be multiple food signals with different degrees of activity.

Exposure of dauers to combined C18 CFA and C18∶1n9 treatment on K12 bacteria suppressed the positive effect of C18∶1n9 alone on K12 (Figure 2D), suggesting that worms can sense the relative ratio of MUFA to CFA. However, in the absence of bacteria we found that C18 CFA could not inhibit the C18∶1n9 induced increase in recovery (Figure 4C), suggesting that under these conditions the worms are primarily responding to MUFA. One explanation for the inhibitory effect of CFA on K12 bacteria could be that exogenous fatty acids accumulate in bacteria and lead to the generation of a secondary metabolite that influences the worm. We did find that saturated, monounsaturated, and cyclopropane fatty acids were detectable in bacterial pellets following 24 h of exposure on NGM plates (Figure S5). In addition, we were able to determine that not only did C18 CFA get into *cfa* bacteria, but it also appeared to be metabolized based upon the appearance of a peak in the gas chromatogram that corresponded to C16 CFA that was absent in the untreated *cfa* bacteria. It is therefore possible that the inhibitory effect of C18 CFA on MUFA-induced recovery on K12 bacteria is due to a secondary effect of the fatty acid on bacterial metabolism. However, the fact that saturated and monounsaturated fatty acids dose-dependently promoted recovery from dauer in both the *daf-2* mutant background in the presence of bacteria and in *daf-2* and wild type dauers in the absence of bacteria shows that the worm is responding to free fatty acids.

Under laboratory conditions, *C. elegans* are maintained in the presence of an abundant supply of food, and thus rarely enter the dauer stage. In contrast, in its natural environment, the worm is likely to experience conditions of fluctuating food availability and dauers are more common [30]. Since β-oxidation of fatty acids yields more ATP per substrate molecule than carbohydrate metabolism, an attractive hypothesis is that by sensing the presence of fatty acids the dauer larva is assessing the energetic value of the food sources in the environment. In this context, it would be beneficial to determine whether sufficient nutrients are available to support growth to the reproductive stage before making the decision to exit the dauer stage.

## Supporting Information

Figure S1 **Exogenous fatty acids influence recovery of** ***daf-2(e1368)*** **dauers on** ***E. coli*** **K12. (A)** Exogenous C16∶1 and C18∶1n7 MUFA augmented dauer recovery on K12 bacteria. (C16∶1 p<0.001 for 0 vs 10 µM and p<0.0001 for 0 vs 25 µM and 0 vs 50 µM; C18∶1n7, p<0.0001 for 0 vs 50 µM)**. (B)** Exogenous C16∶0 and C18∶0 saturated fatty acids augmented dauer recovery on K12 bacteria. (C16∶0 p<0.0001 for 0 vs 25 µM and 0 vs 50 µM; C18∶0 p<0.0001 for 0 vs 10 µM, 0 vs 25 µM and 0 vs 50 µM)**. (C)** Neither *cfa* bacteria nor exogenous C18∶1n9 promoted dauer recovery in *daf-2(e1368)* dauers after 24 h at 25°C.
(DOCX)

Figure S2 **Dauer recovery increases on** ***cfa*** **bacteria and in response to exogenous C18∶1n9 in other alleles of** ***daf-2.*** **(A)** Dauer recovery was enhanced on *cfa* bacteria compared with K12 for each allele of *daf-2*. **(B)** Dauer recovery was enhanced on K12 bacteria supplemented with 50 µM C18∶1n9 for each allele of *daf-2*. (****p<0.0001 for indicated pairwise comparisons).
(DOCX)

Figure S3 **Non-transgenic siblings from ASJ ablation strains remain responsive to fatty acids. (A)** *cfa* bacteria promoted dauer recovery in the non-transgenic siblings of *daf-2(e1368)* dauers in which the ASJ sensory neuron was genetically ablated (*daf-2; jxEx102* and *daf-2; jxEx100*) compared with the control line (*daf-2; jxEx18).* **(B)** Supplementation of K12 bacteria with 50 µM C18∶1n9 promoted dauer recovery in the non-transgenic siblings of *daf-2(e1368)* dauers in which the ASJ sensory neuron was genetically ablated (*daf-2; jxEx102* and *daf-2; jxEx100*) compared with the control line (*daf-2; jxEx18).* (****p<0.0001 for indicated pairwise comparisons).
(DOCX)

Figure S4 **Recovery of** ***daf-2(e1368)*** **dauers at 20°C supplemented with fatty acids in the absence of bacteria. (A)** Exogenous C16∶0, C16∶1 and C18∶1n7 promoted recovery of *daf-2(e1368)* dauers in the absence of bacteria. (C16∶0 p<0.001 for 0 vs 2.5 µM and p<0.0001 for 0 vs 5 µM, 0 vs 10 µM and 0 vs 25 µM; C16∶1 p<0.0001 for 0 vs 5 µM, 0 vs 10 µM and 0 vs 25 µM; C18∶1n7 p<0.0001 for 0 vs 5 µM, 0 vs 10 µM and 0 vs 25 µM). **(B)** Other saturated and monounsaturated fatty acids, but not polyunsaturated fatty acids, promoted dauer recovery in the absence of bacteria. (*p<0.05 vs control, ****p<0.0001 vs control).
(DOCX)

Figure S5 **Fatty acid profiles in** ***E.coli*** **K12 and** ***cfa*** **following exposure to exogenous fatty acids. (A)** Fatty acid profiles in K12 bacteria following exposure to 50 µM C18∶1n9 or C18∶0 on NGM plates. The amount of each bacterial fatty acid is expressed as a percent of the total area under the curve. C18∶1n9 was not present in untreated K12 but comprised 4% of total fatty acids after exposure to 50 µM C18∶1n9. Treatment with 50 µM C18∶0 increased bacterial C18∶0 from 1.4% to 16.4%. **(B)** Fatty acid profiles in *cfa* bacteria following exposure to 50 µM C18 CFA on NGM plates. C18 and C16 CFA were undetectable in *cfa* bacteria but constituted 1.6% and 2.4% of the total fatty acids respectively after treatment with C18 CFA. Data are presented as mean+sd for 8 independent bacterial samples. NGM plates spotted with K12 or *cfa* ±50 µM C18∶1n9, C18∶0 or C18 CFA were incubated at 20°C for 24 h. The bacteria were washed off the plate with 1 mL S-basal into a microcentrifuge tube. After centrifugation, the supernatant was discarded and the pellet was washed with 1 mL S-basal. This was repeated such that the pellet was washed 5 times to remove exogenous fatty acids before FAME analysis was carried out as described in the methods.
(DOCX)

Table S1 Raw data for dauer recovery experiments.
(XLSX)
