# Supplementary material for: Bacterial Fatty Acids Enhance Recovery from the Dauer Larva in Caenorhabditis elegans
Source: PLoS One. 2014 Jan 24;9(1):e86979. doi: 10.1371/journal.pone.0086979 (PMC3901721; doi:10.1371/journal.pone.0086979)
Supplement: Figure S1 — Exogenous fatty acids influence recovery of daf-2(e1368) dauers on E. coli K12. (A) Exogenous C16∶1 and C18∶1n7 MUFA augmented dauer recovery on K12 bacteria. (C16∶1 p<0.001 for 0 vs 10 µM and p<0.0001 for 0 vs 25 µM and 0 vs 50 µM; C18∶1n7, p<0.0001 for 0 vs 50 µM). (B) Exogenous C16∶0 and C18∶0 saturated fatty acids augmented dauer recovery on K12 bacteria. (C16∶0 p<0.0001 for 0 vs 25 µM and 0 vs 50 µM; C18∶0 p<0.0001 for 0 vs 10 µM, 0 vs 25 µM and 0 vs 50 µM). (C) Neither cfa bacteria nor exogenous C18∶1n9 promoted dauer recovery in daf-2(e1368) dauers after 24 h at 25°C. (DOCX) [file pone.0086979.s001.docx]

## Figure S1: Exogenous fatty acids influence recovery of *daf-2(e1368)* dauers on *E. coli* K12.

**A**  **B**

**C**

**(A)** Exogenous C16:1 and C18:1n7 MUFA augmented dauer recovery on K12 bacteria. (C16:1 p<0.001 for 0 vs 10 µM and p<0.0001 for 0 vs 25 µM and 0 vs 50 µM; C18:1n7, p<0.0001 for 0 vs 50 µM)**. (B)** Exogenous C16:0 and C18:0 saturated fatty acids augmented dauer recovery on K12 bacteria. (C16:0 p<0.0001 for 0 vs 25 µM and 0 vs 50 µM; C18:0 p<0.0001 for 0 vs 10 µM, 0 vs 25 µM and 0 vs 50 µM)**. (C)** Neither *cfa* bacteria nor exogenous C18:1n9 promoted dauer recovery in *daf-2(e1368)* dauers after 24h at 25°C.
