# Supplementary material for: Bacterial Fatty Acids Enhance Recovery from the Dauer Larva in Caenorhabditis elegans
Source: PLoS One. 2014 Jan 24;9(1):e86979. doi: 10.1371/journal.pone.0086979 (PMC3901721; doi:10.1371/journal.pone.0086979)
Supplement: Figure S2 — Dauer recovery increases on cfa bacteria and in response to exogenous C18∶1n9 in other alleles of daf-2. (A) Dauer recovery was enhanced on cfa bacteria compared with K12 for each allele of daf-2. (B) Dauer recovery was enhanced on K12 bacteria supplemented with 50 µM C18∶1n9 for each allele of daf-2. (****p<0.0001 for indicated pairwise comparisons). (DOCX) [file pone.0086979.s002.docx]

## Figure S2: Dauer recovery increases on *cfa* bacteria and in response to exogenous C18:1n9 in other alleles of *daf-2*

**A B**

**(A)** Dauer recovery was enhanced on *cfa* bacteria compared with K12 for each allele of *daf-2*. **(B)** Dauer recovery was enhanced on K12 bacteria supplemented with 50 µM C18:1n9 for each allele of *daf-2*. (****p <0.0001 for indicated pairwise comparisons).
