# Supplementary material for: Bacterial Fatty Acids Enhance Recovery from the Dauer Larva in Caenorhabditis elegans
Source: PLoS One. 2014 Jan 24;9(1):e86979. doi: 10.1371/journal.pone.0086979 (PMC3901721; doi:10.1371/journal.pone.0086979)
Supplement: Figure S3 — Non-transgenic siblings from ASJ ablation strains remain responsive to fatty acids. (A) cfa bacteria promoted dauer recovery in the non-transgenic siblings of daf-2(e1368) dauers in which the ASJ sensory neuron was genetically ablated (daf-2; jxEx102 and daf-2; jxEx100) compared with the control line (daf-2; jxEx18). (B) Supplementation of K12 bacteria with 50 µM C18∶1n9 promoted dauer recovery in the non-transgenic siblings of daf-2(e1368) dauers in which the ASJ sensory neuron was genetically ablated (daf-2; jxEx102 and daf-2; jxEx100) compared with the control line (daf-2; jxEx18). (****p<0.0001 for indicated pairwise comparisons). (DOCX) [file pone.0086979.s003.docx]

## Figure S3: Non-transgenic siblings from ASJ ablation strains remain responsive to fatty acids

**A B**

**(A)** *cfa* bacteria promoted dauer recovery in the non-transgenic siblings of *daf-2(e1368)* dauers in which the ASJ sensory neuron was genetically ablated (*daf-2; jxEx102* and *daf-2; jxEx100*) compared with the control line (*daf-2; jxEx18).* **(B)** Supplementation of K12 bacteria with 50 µM C18:1n9 promoted dauer recovery in the non-transgenic siblings of *daf-2(e1368)* dauers in which the ASJ sensory neuron was genetically ablated (*daf-2; jxEx102* and *daf-2; jxEx100*) compared with the control line (*daf-2; jxEx18).*  (****p <0.0001 for indicated pairwise comparisons).
