# Supplementary material for: Bacterial Fatty Acids Enhance Recovery from the Dauer Larva in Caenorhabditis elegans
Source: PLoS One. 2014 Jan 24;9(1):e86979. doi: 10.1371/journal.pone.0086979 (PMC3901721; doi:10.1371/journal.pone.0086979)
Supplement: Figure S4 — Recovery of daf-2(e1368) dauers at 20°C supplemented with fatty acids in the absence of bacteria. (A) Exogenous C16∶0, C16∶1 and C18∶1n7 promoted recovery of daf-2(e1368) dauers in the absence of bacteria. (C16∶0 p<0.001 for 0 vs 2.5 µM and p<0.0001 for 0 vs 5 µM, 0 vs 10 µM and 0 vs 25 µM; C16∶1 p<0.0001 for 0 vs 5 µM, 0 vs 10 µM and 0 vs 25 µM; C18∶1n7 p<0.0001 for 0 vs 5 µM, 0 vs 10 µM and 0 vs 25 µM). (B) Other saturated and monounsaturated fatty acids, but not polyunsaturated fatty acids, promoted dauer recovery in the absence of bacteria. (*p<0.05 vs control, ****p<0.0001 vs control). (DOCX) [file pone.0086979.s004.docx]

## Figure S4: Recovery of *daf-2(e1368)* dauers at 20°C supplemented with fatty acids in the absence of bacteria.

**A B**

**(A)** Exogenous C16:0, C16:1 and C18:1n7 promoted recovery of *daf-2(e1368)* dauers in the absence of bacteria. (C16:0 p<0.001 for 0 vs 2.5 µM and p<0.0001 for 0 vs 5 µM, 0 vs 10 µM and 0 vs 25 µM; C16:1 p<0.0001 for 0 vs 5 µM, 0 vs 10 µM and 0 vs 25 µM; C18:1n7 p<0.0001 for 0 vs 5 µM, 0 vs 10 µM and 0 vs 25 µM). **(B)** Other saturated and monounsaturated fatty acids, but not polyunsaturated fatty acids, promoted dauer recovery in the absence of bacteria. (* p<0.05 vs control, ****p<0.0001 vs control).
