# Supplementary material for: Bacterial Fatty Acids Enhance Recovery from the Dauer Larva in Caenorhabditis elegans
Source: PLoS One. 2014 Jan 24;9(1):e86979. doi: 10.1371/journal.pone.0086979 (PMC3901721; doi:10.1371/journal.pone.0086979)
Supplement: Figure S5 — Fatty acid profiles in E.coli K12 and cfa following exposure to exogenous fatty acids. (A) Fatty acid profiles in K12 bacteria following exposure to 50 µM C18∶1n9 or C18∶0 on NGM plates. The amount of each bacterial fatty acid is expressed as a percent of the total area under the curve. C18∶1n9 was not present in untreated K12 but comprised 4% of total fatty acids after exposure to 50 µM C18∶1n9. Treatment with 50 µM C18∶0 increased bacterial C18∶0 from 1.4% to 16.4%. (B) Fatty acid profiles in cfa bacteria following exposure to 50 µM C18 CFA on NGM plates. C18 and C16 CFA were undetectable in cfa bacteria but constituted 1.6% and 2.4% of the total fatty acids respectively after treatment with C18 CFA. Data are presented as mean+sd for 8 independent bacterial samples. NGM plates spotted with K12 or cfa ±50 µM C18∶1n9, C18∶0 or C18 CFA were incubated at 20°C for 24 h. The bacteria were washed off the plate with 1 mL S-basal into a microcentrifuge tube. After centrifugation, the supernatant was discarded and the pellet was washed with 1 mL S-basal. This was repeated such that the pellet was washed 5 times to remove exogenous fatty acids before FAME analysis was carried out as described in the methods. (DOCX) [file pone.0086979.s005.docx]

## Figure S5: Fatty acid profiles in *E.coli* K12 and *cfa* following exposure to exogenous fatty acids

**A B**

**(A)** Fatty acid profiles in K12 bacteria following exposure to 50 µM C18:1n9 or C18:0 on NGM plates. The amount of each bacterial fatty acid is expressed as a percent of the total area under the curve. C18:1n9 was not present in untreated K12 but comprised 4% of total fatty acids after exposure to 50 µM C18:1n9. Treatment with 50 µM C18:0 increased bacterial C18:0 from 1.4% to 16.4%. **(B)** Fatty acid profiles in *cfa* bacteria following exposure to 50 µM C18 CFA on NGM plates. C18 and C16 CFA were undetectable in *cfa* bacteria but constituted 1.6% and 2.4% of the total fatty acids respectively after treatment with C18 CFA. Data are presented as mean+sd for 8 independent bacterial samples. NGM plates spotted with K12 or *cfa* ± 50 µM C18:1n9, C18:0 or C18 CFA were incubated at 20⁰C for 24 h. The bacteria were washed off the plate with 1 mL S-basal into a microcentrifuge tube. After centrifugation, the supernatant was discarded and the pellet was washed with 1 mL S-basal. This was repeated such that the pellet was washed 5 times to remove exogenous fatty acids before FAME analysis was carried out as described in the methods.
